# Structure of a microtubule-bound axonemal dynein

**DOI:** 10.1038/s41467-020-20735-7

**Published:** 2021-01-20

**Authors:** Travis Walton, Hao Wu, Alan Brown

**Affiliations:** Department of Biological Chemistry and Molecular Pharmacology, Blavatnik Institute, Harvard Medical School, 240 Longwood Avenue, Boston, MA USA

**Keywords:** Cryoelectron microscopy, Cilia, Dynein

## Abstract

Axonemal dyneins are tethered to doublet microtubules inside cilia to drive ciliary beating, a process critical for cellular motility and extracellular fluid flow. Axonemal dyneins are evolutionarily and biochemically distinct from cytoplasmic dyneins that transport cargo, and the mechanisms regulating their localization and function are poorly understood. Here, we report a single-particle cryo-EM reconstruction of a three-headed axonemal dynein natively bound to doublet microtubules isolated from cilia. The slanted conformation of the axonemal dynein causes interaction of its motor domains with the neighboring dynein complex. Our structure shows how a heterotrimeric docking complex specifically localizes the linear array of axonemal dyneins to the doublet microtubule by directly interacting with the heavy chains. Our structural analysis establishes the arrangement of conserved heavy, intermediate and light chain subunits, and provides a framework to understand the roles of individual subunits and the interactions between dyneins during ciliary waveform generation.

## Abstract (web summary)

Axonemal dyneins are tethered to doublet microtubules inside cilia to drive ciliary beating but the mechanisms regulating their localization and function are poorly understood. Here authors report a cryo-EM reconstruction of a three-headed axonemal dynein natively bound to doublet microtubules isolated from cilia which provides a framework to understand the roles of individual subunits.

## Introduction

Dyneins are a eukaryotic family of ATP-dependent microtubule motor^1^. They are categorized by their two major roles inside cells; cytoplasmic dyneins travel along microtubules and are responsible for both intracellular and intraflagellar transport^2^, whereas axonemal dyneins are docked onto doublet microtubules inside cilia and power ciliary beating^3^. The coordinated activity of thousands of axonemal dyneins bends doublet microtubules to generate the ciliary waveform, which has evolved to efficiently displace fluid, allowing either cell self-propulsion or the flow of extracellular liquid over epithelial surfaces. In humans, ciliary motility is responsible for diverse biological processes, including symmetry breaking during embryogenesis^4^, the circulation of cerebrospinal fluid in the brain^5^, mucociliary clearance in innate defense^6^, and the swimming of spermatozoa^7^.

The axoneme of most motile cilia consists of a circular array of doublet microtubules^8^. The axonemal dyneins are attached to the doublet microtubules in two continuous rows known as the outer dynein arm (ODA) and the inner dynein arm (IDA). The dyneins within these arms have distinct molecular compositions, periodicities, and roles in ciliary waveform generation^9,10^, although each generates force by pushing against their neighboring doublet microtubule^11^. The ODA has a single large dynein motor that repeats every 24 nm and influences the frequency of ciliary waveforms^9,10^. In contrast, the IDA regulates the amplitude of ciliary waveforms and is formed by seven smaller dyneins (IDA*a-g*) that each repeat every 96 nm^12^. Genetic studies in the model organism *Chlamydomonas reinhardtii* have revealed that loss of ODAs causes more severe defects in ciliary motility than the loss of individual IDAs^13,14^. In humans, mutations in ODA subunits are associated with male infertility^15^ and are the major cause of primary ciliary dyskinesia (PCD)^16,17^, a congenital disease characterized by chronic airway infection and laterality abnormalities.

All dynein motors have at least one heavy chain, which contains the force-generating AAA+ motor domain. Cytoplasmic dyneins have two identical heavy chains^2^, whereas ODAs can have up to three different heavy chains. These heavy chains are assembled with light and intermediate chain subunits to form the functional ODA complex^18^. Each ODA complex attaches to doublet microtubules through a docking complex, the ODA-DC^19^. A partial structure for a heterotrimeric ODA-DC has been determined bound to native doublet microtubules of *C. reinhardtii* by single-particle cryo-electron microscopy (cryo-EM)^20^. However, the structure of the ODA has only been observed by low-resolution cryo-electron tomography (cryo-ET)^21–24^. To understand the unique subunit organization of axonemal dyneins and molecular mechanisms that regulate their function, we have determined a structure of the ODA complex in its microtubule-bound post-powerstroke state using single-particle cryo-EM.

## Results

### Structure determination of ODA-bound doublet microtubules

Previous cryo-EM structures of doublet microtubules have all lacked ODAs^20,25^. To generate a sample that retained ODAs, we, therefore, took an alternative approach and used incubation with ATP and calcium to fray or splay *C. reinhardtii* axonemes^26,27^ rather than ATP and protease treatment to fully dissociate them (Supplementary Fig. 1a, b). Negative-stain EM of the splayed axonemes revealed clusters of ODA complexes bound to doublet microtubules with characteristic 24-nm periodicity (Supplementary Fig. 1c). This axoneme-splaying procedure was then optimized to generate samples for cryo-EM (Supplementary Fig. 2a).

The cryo-EM data were initially processed to reconstruct the 24-nm repeat of the doublet microtubule, excluding all particles that lacked ODAs (Supplementary Fig. 2b, c). We then refocused and recentered our refinement on protofilaments A06-A08, to which ODA is bound. This reference map provides the overall structure of the ODA and reveals the structural basis of docking to the ODA-DC. Although the nominal resolution is 3.8 Å (Supplementary Fig. 3a), the local resolution varies considerably (Supplementary Fig. 3b). We, therefore, performed a series of focused refinements to improve the map quality, with the core resolved to 3.7 Å (Supplementary Figs. 3c–f and 4). These local maps and their merged composites (Supplementary Table 1) allowed us to place models of all three heavy chains (α-HC, β-HC, and γ-HC), both intermediate chains (IC1 and IC2), 8 of the 11 proposed light chains^18,28^, and all 3 subunits of the ODA-DC (Supplementary Movie 1 and Supplementary Table 2). We cannot resolve LC1, which is bound to the microtubule-binding domain (MTBD) of γ-HC^29^ or the thioredoxin-like proteins LC3 and LC5. Model statistics are provided in Supplementary Table 3.

### Cooperative assembly through inter-dynein contacts

**Fig. 1:**
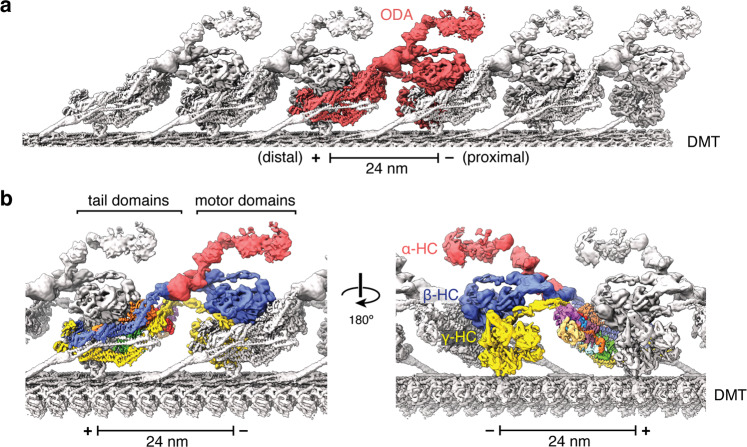
Structure of the ODA axonemal dynein bound to a doublet microtubule. **a** Composite map showing the 24-nm repeat of the ODA bound to protofilaments A07 and A08 of the doublet microtubule (DMT). A single ODA complex (colored red) forms an elongated and inclined structure that interacts with both its neighbors. **b** Two views showing the ODA complex colored by subunit. The motor domains form a triple stack in which the motors of both β- and γ-HC interact with the tail domains of the proximal ODA. The minus (−) and plus (+) ends of the microtubule are indicated on the scale bar.

The three AAA+ domains of the ODA are stacked in parallel, with the motor domain of the γ-HC closest to the doublet microtubule surface but not in contact with it, and α-HC furthest away (Fig. 1). Stripping the ODA complex from doublet microtubules typically results in dissociation of γ-HC^28^. Since our sample retained all three heavy chains in a conformation similar to that seen by cryo-ET of axonemes^21^, this structure likely represents a native complex retained on doublet microtubules. However, we cannot completely exclude the possibility of small conformational changes to the ODA in splayed versus intact axonemes, especially in regions far from the microtubule surface.

Each dynein adopts an extended and slanted configuration on the doublet microtubule in which the motor domains of β-HC and γ-HC closely associate with the heavy chain tail domains and auxiliary subunits of the preceding (proximal) ODA (Fig. 1a, b). This confirms the “overlap” model first proposed by Goodenough and Heuser^30^, in which the large 24-nm globular repeat of the ODA is actually a combination of neighboring axonemal dyneins. The interactions between neighboring complexes may help explain why ODAs form a continuous, linear array on doublet microtubules and why ODAs appear in clusters during disassembly (Supplementary Fig. 1b, c) or during reconstitution on axonemes^31^. Mutants lacking the AAA+ motor domains of β-HC and γ-HC, which would also reduce inter-dynein contacts, have a decreased amount of ODAs bound to the axoneme, while the α-HC mutant does not display this phenotype^32–34^.

### Motor domains in post-powerstroke conformation

**Fig. 2:**
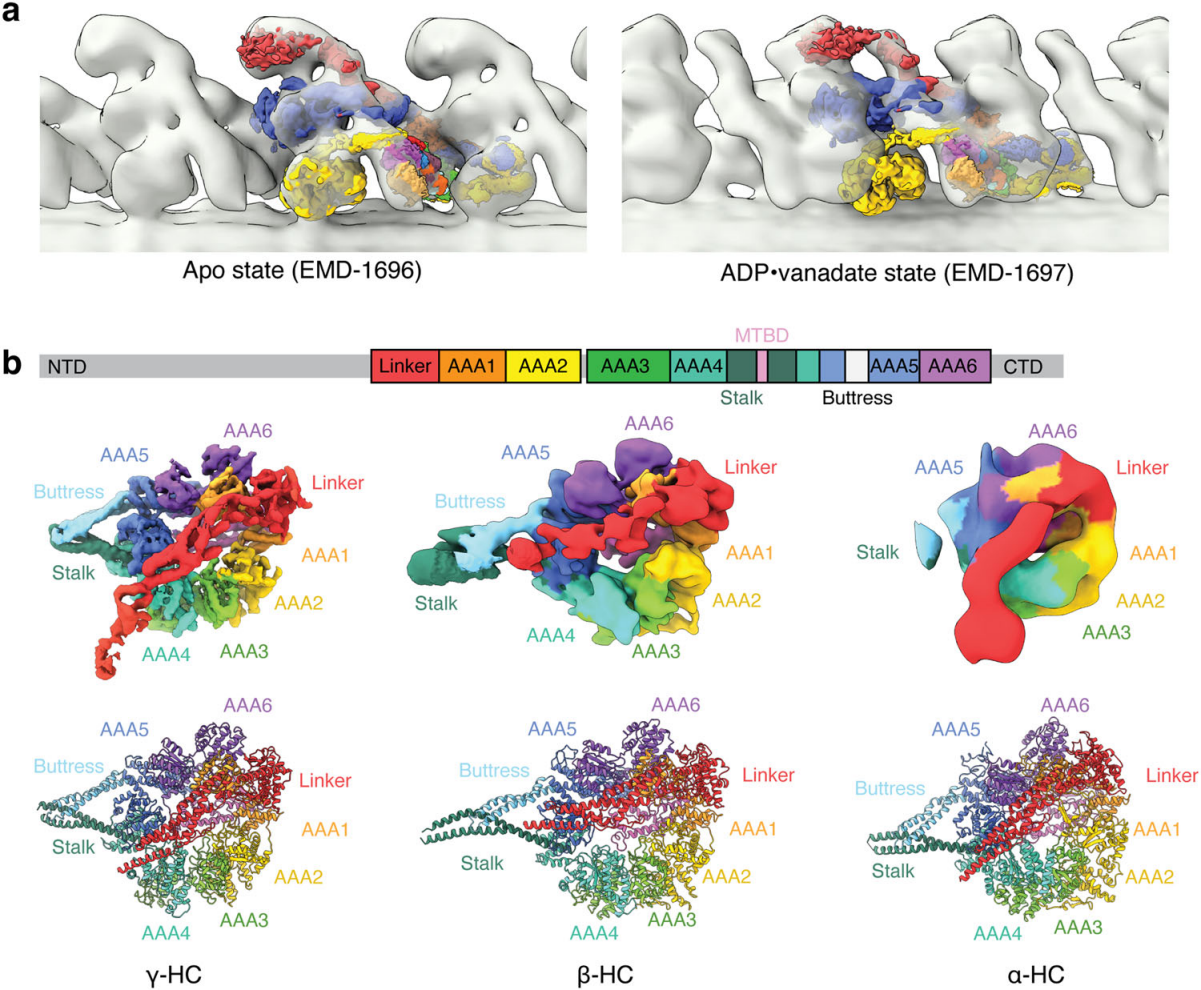
The ODA complexes are in an apo state. **a** The composite map of the ODA generated from our single-particle cryo-EM approach was docked into the subtomogram volumes of the *C. reinhardtii* ODA in apo and ADP•Vanadate states (representing the post- and pre-powerstroke states, respectively). The map fits well into the apo state. **b** General domain organization of an axonemal dynein heavy chain. Density maps and models of the motor domains of the three heavy chains colored by subdomain. The linker of β-HC adopts a different angle relative to the stalk compared to the linkers of α- and γ-HC.

Based on comparison with cryo-ET reconstructions in different nucleotide states^21,24^, all heavy chains appear to be in the apo or ADP-bound conformation (Fig. 2a, Supplementary Movie 2), oriented for minus-end directed movement. The motor domains show considerable flexibility relative to the rest of the complex. Using three-dimensional classification and mask-focused refinement, we generated individual maps of each motor domain and superposed these onto the reference (Supplementary Fig. 3c). The γ-HC motor domain is resolved to ~4.5 Å, two conformations of the β-HC motor domain are resolved to ~6 and ~10 Å, and the α-HC motor domain to ~12 Å. Consistent with the motors being in an apo state, we do not observe density for nucleotides in the highest resolution γ-HC map. The decrease in resolution with distance from the doublet microtubule surface indicates that the more distal motors have greater freedom to move in the splayed axoneme samples. In intact axonemes, the conformations of the motor domains are likely stabilized by interactions with the neighboring doublet microtubule.

The maps allow confident placement of the linker region of the dynein tail, the AAA+ ring, and the proximal region of the buttress-stabilized coiled-coil stalk. The distal MTBD is not observed, presumably due to the flexibility of the stalk. For all three heavy chains, the linker is in a straight conformation and docked onto the AAA5 module (Fig. 2b), features characteristic of dynein motor domains in the post-powerstroke state^35^. The linker is bent and associates with AAA3 in the pre-powerstroke (priming) state following ATP hydrolysis. Relative to α-HC and γ-HC, the linker for β-HC lies closer to the stalk in the majority of our particles. The consequence of this difference is that the stalk of β-HC does not point outwards in the direction of the B tubule like the stalks of α-HC and γ-HC but is directed back and interacts with the helical bundles of its own tail (Supplementary Fig. 5a). A second class in which the β-HC stalk points outwards, parallel with the stalks of α-HC and γ-HC (Supplementary Figs. 3c and 5b), indicates that the β-HC motor domain exists in multiple states that are distinct from the pre-powerstroke state^21^.

In our observed apo state, the γ-HC and β-HC motor domains make inter-dynein contacts with the proximal dynein and intra-dynein contacts with one another through their linker domains (Supplementary Fig. 6 and Supplementary Movie 3). The C-terminal region of the linker of the γ-HC motor domain contacts helical bundle 3 of the proximal copy of the γ-HC tail, whereas the same region of the β-HC linker interacts with IC2. The middle of the γ-HC linker forms inter-motor contacts with the α-helical subdomain of β-HC AAA5. This network of interactions involving the linker may have a regulatory role, and must change in the pre-powerstroke state, with the motor domains forming even closer associations with the proximal dynein (Fig. 2a)^21^.

### Heterotrimerization of dynein motor tail domains

All three heavy chains are connected to each other through their N-terminal tail domains (Supplementary Fig. 7). The tails of β-HC and γ-HC are architecturally similar to the tails of cytoplasmic dyneins, consisting of an N-terminal dimerization domain (NDD) and a series of α-helical bundles connected in a zig-zag configuration. The NDDs of β-HC and γ-HC form a heterodimer (Supplementary Fig. 7c) that is similar to the homodimeric NDDs of dynein-1 and dynein-2 and is located near the ODA-DC at the doublet microtubule surface^36,37^. In contrast, the α-HC attaches to the outside of helical bundle 6 of β-HC through an N-terminal, kelch-type six-stranded β-propeller that is located 170 Å away from the NDD of β-HC and γ-HC (Supplementary Fig. 7d). This site of attachment positions α-HC away from the microtubule surface and peripheral to other ODA subunits. This conformation is consistent with observations that loss of α-HC in the *Chlamydomonas* mutant *oda-11* results in a semi-functional ODA complex containing just β- and γ-HCs^32^. Notably, α-HC is not conserved in metazoans, including humans, with two-headed ODAs^38^. Our results help explain how the conventional two-headed structure of dyneins can be modified to include a third head, and why the loss of the third head through evolution or mutation does not lead to loss of the entire ODA complex.

**Fig. 3:**
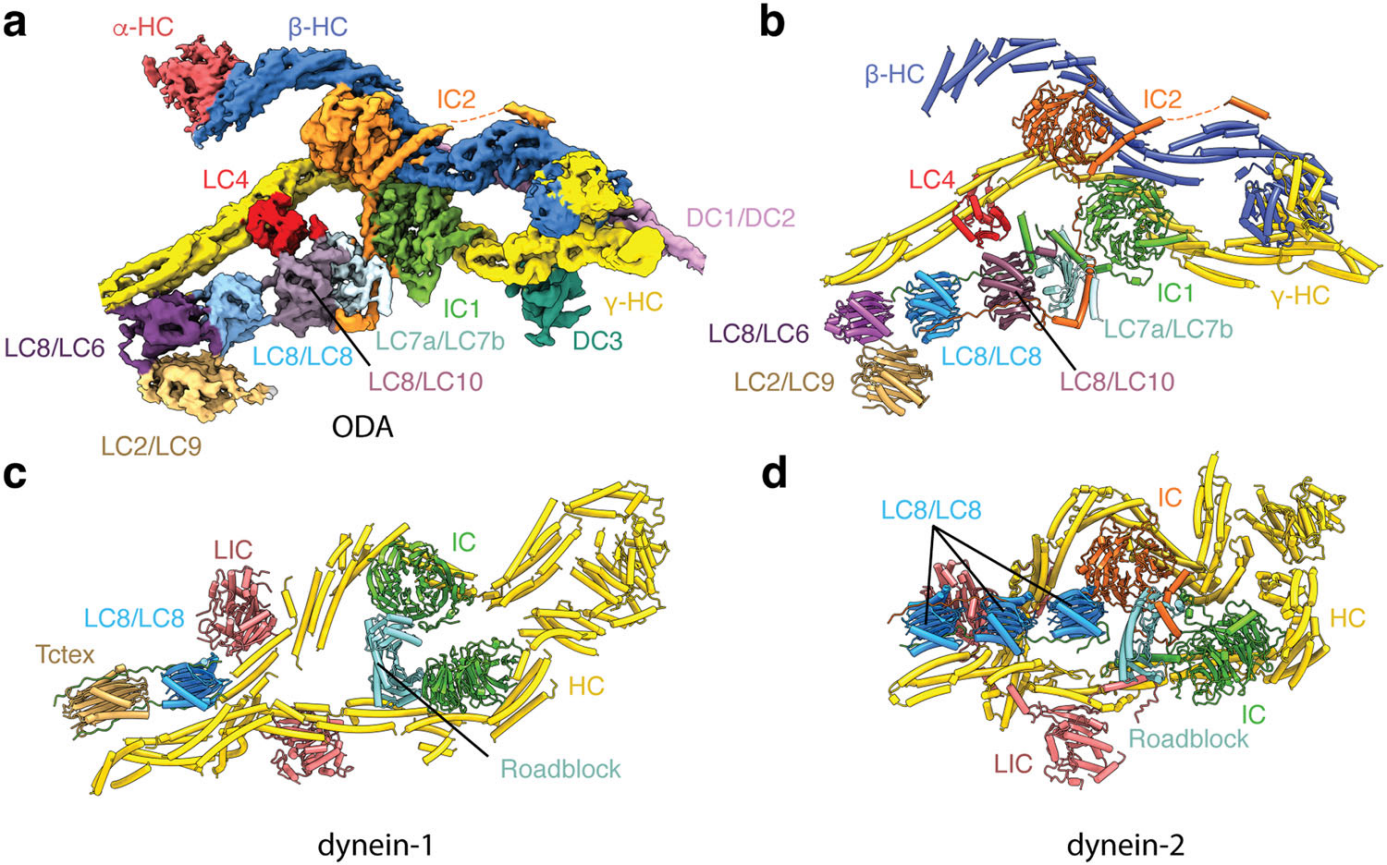
Arrangement of the HC tails and IC–LC block and comparison with cytoplasmic dyneins. **a** Map of the IC–LC block of the native *C. reinhardtii* ODA complex colored by subunit. **b** Atomic model of the IC–LC block of the *C. reinhardtii* ODA complex colored by subunit. **c** Atomic model of the IC–LC block of recombinant human dynein-1 (PDB 5NVU)^39^. **d** Atomic model of the IC–LC block of recombinant human dynein-2 (PDB 6RLB)^37^.

The conformations of the β- and γ-HC tails are supported by a “block” of intermediate and light chains that resembles a similar subcomplex found in cytoplasmic dyneins^37,39^ (Fig. 3 and Supplementary Movie 1). This block contains two intermediate chains (IC1 and IC2), each with a β-propeller domain that interacts with both β- and γ-HC. The extended N-termini of the intermediate chains are bound by a heterodimer of the Roadblock homologs LC7a and LC7b, three different members of the LC8 family existing as homo- and heterodimers (LC6/LC8/LC10; Supplementary Fig. 4b), and a presumed heterodimer of TCTEX homologs LC2 and LC9. These light chains interact with γ-HC, but not α- or β-HC. Cytoplasmic dyneins lack the additional LC8 paralogs and have either one (dynein-1) or three copies (dynein-2) of the LC8 homodimer (Fig. 3c, d). Thus, axonemal and cytoplasmic dyneins have similar arrangements of auxiliary factors implying that they share conserved regulatory mechanisms.

### Structural basis of docking to doublet microtubules

**Fig. 4:**
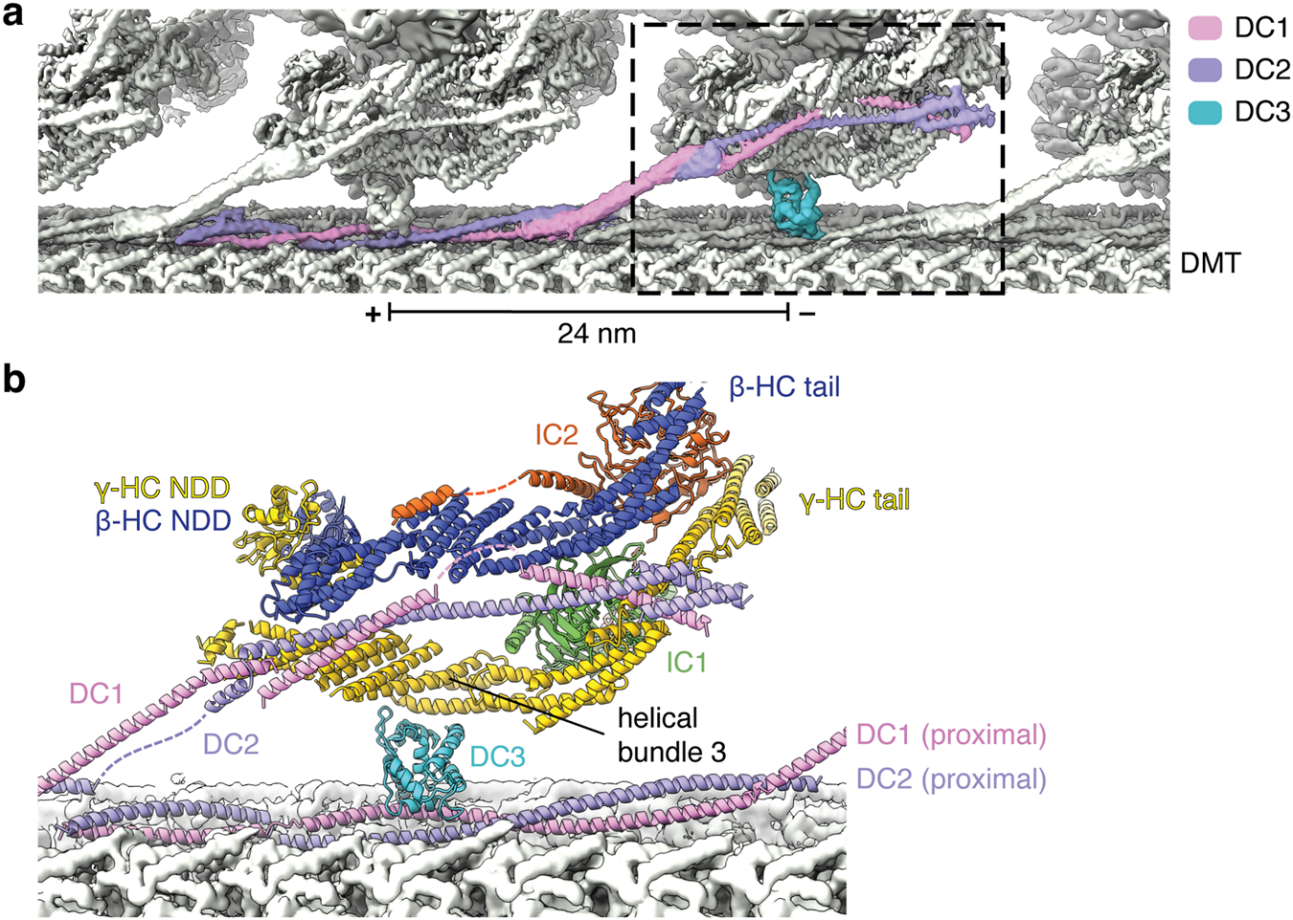
Structural basis for the docking of axonemal dyneins to the doublet microtubule surface. **a** The ODA docking complex (ODA-DC) is a heterotrimer consisting of a DC1/DC2 coiled-coil and a globular DC3 subunit that repeats every 24 nm. Each ODA complex interacts with one copy of DC1/DC2 coiled-coil, and the DC3 subunit from the proximal ODA-DC. Thus, each ODA-DC contributes to the binding of two ODA complexes. **b** Atomic model of the interactions between the ODA and the ODA-DC. The C terminus of the DC1/DC2 coiled-coil interacts with the tail domains of β- and γ-HC. DC3 interacts with helical bundle 3 of the γ-HC tail. NDD N-terminal dimerization domain.

In *Chlamydomonas*, the ODA-DC is a heterotrimer made of a coiled-coil of DC1 and DC2 and a calcium-binding subunit, DC3^19,20^. Our previous structure of a doublet microtubule revealed that the N-terminal half of the DC1/DC2 coiled-coil occupies the external cleft between protofilaments A07 and A08^20^. In the ODA-bound state, DC3 sits midway along the microtubule-bound portion of the DC1/2 coiled-coil and interacts exclusively with helical bundle 3 of the γ-HC (Fig. 4b). The C-terminal halves of DC1/2 are also resolved and elevate from the surface of the doublet microtubule to interact with the neighboring proximal ODA. They bisect the tails of β-HC and γ-HC, making contacts with both (Fig. 4), and span as far as the ICs, explaining the ability of both ICs to form chemical crosslinks with DC1 and DC2^40^. Thus, the tails of β- and γ-HC are required for the attachment of axonemal dyneins to doublet microtubules. This explains why truncated β-HC and γ-HC mutants that retain their N-termini but lack a C-terminal motor domain still form ODAs with partial activity^33,34^.

The ODA is tethered to doublet microtubules almost exclusively through the ODA-DC, explaining why mutations in ODA-DC subunits are associated with decreased levels or complete loss of ODA complexes from doublet microtubules^19,41^. We do not observe any interactions between IC1 and the doublet microtubule, even though IC1 crosslinks with α-tubulin and binds microtubules in vitro^40,42,43^. Of the two regions of IC1 previously shown to bind microtubules, the N-terminal 20 amino acids are not resolved in our final cryo-EM maps, and thus might form a flexible connection to the microtubule surface. The second region (D264–D323) forms a well-resolved part of the β-propeller domain and abutting α-helix. Thus, this region is unlikely to bind microtubules in the post-powerstroke conformation of the ODA.

### A calcium-responsive, ODA-specific light chain

Calcium is an important regulator of the ciliary beat^44^ and can directly alter the waveform of isolated *Chlamydomonas* axonemes in vitro, causing a switch from a normal asymmetric beat to a symmetric beat at Ca^2+^ concentrations between 10 and 100 μM^45^. This calcium-dependent switch in waveforms occurs in vivo during photoshock (sudden exposure to bright light). As ODA mutants lack a photoshock response^41^, it has been suggested that a calcium sensor resides within axonemal dyneins. Previous studies have proposed that a Ca^2+^-responsive element in ODAs is LC4, a calmodulin-like subunit with three EF-hands that is specific to axonemal dyneins. LC4 binds Ca^2+^ in vitro with a *K*_Ca_ of ~25 μM^46^ and undergoes Ca^2+^-dependent interactions with both γ-HC and IC1^47^. Furthermore, when a complex of γ-HC and LC4 is treated with Ca^2+^, the N-terminal tail of γ-HC bends, suggesting LC4 is directly able to modulate the dynamics of the γ-HC in a calcium-dependent manner^47^.

Our structure shows LC4 bound at the junction between helical bundles 6 and 7 of the γ-HC tail (Fig. 3a and Supplementary Fig. 8). In cytoplasmic dyneins, this junction is usually occupied by a light intermediate chain (LIC)^37,39^ that is absent from ODAs. LC4 has been previously shown to interact with IC1 in the presence of calcium ions^47^, and our structure shows the N-terminal half of LC4 in close proximity to the N-terminal extensions of IC1 (Supplementary Fig. 8b). Notably, our sample contains 250 μM-free Ca^2+^, and the helix–loop–helix angles of these EF-hands approach 90°, which is consistent with the Ca^2+^-bound state of other calmodulin-like proteins, although our map quality is insufficient to resolve bound cations. In contrast, the C-terminal half of LC4 contains one EF-hand and one helix–loop–helix with no discernible Ca^2+^-binding motif. Binding of LC4 to the helical bundles does not involve the two predicted IQ motifs on γ-HC^47^. We predict that a similar interaction occurs between the calmodulin-like light chain centrin and the helical bundles of IDA *b*, *e*, and *g*^48^ to mediate ciliary waveform regulation by Ca^2+^.

## Discussion

Here, we report a single-particle cryo-EM reconstruction of three-headed axonemal dynein natively bound to doublet microtubules. The structure reveals that β-HC, γ-HC, and the IC–LC block forms a core structure surprisingly similar to cytoplasmic dyneins. This core is expanded by an additional heavy chain (α-HC) appended to the tail of β-HC and the calcium-responsive light chain LC4 bound to the tail of γ-HC. The other axonemal-specific light chains, LC3 and LC5, may interact with the heavy chain tails preceding the motor linkers where our map is least well-resolved^49^.

We show that neighboring dyneins overlap, with the motor domains of β-HC and γ-HC interacting with the IC–LC block and N-terminal tails of the equivalent proteins in the preceding dynein. Due to the limited resolution of the motor domains, we cannot yet elucidate the exact nature of inter-ODA contacts, and whether they result from the steric constraints of periodic docking, or from defined binding sites. However, we propose that inter-ODA contacts could partially explain the cooperative binding of ODAs on doublet microtubules, which has also been observed in the absence of a complete docking complex^50^. The contacts between neighboring dyneins must change during the powerstroke cycle of dyneins, and suggests a putative mechanism for coordinated dynein activity, where an ATP-dependent conformational change in one dynein is propagated to the neighboring dynein. Cryo-ET data have shown that although individual dyneins can exist in different conformational states along the length of a doublet microtubule, clusters of neighboring ODAs are often in the same functional state^21^. The coordinated activity of clusters of dyneins would generate additional force^36^ needed to bend doublet microtubules and generate the ciliary waveform.

Despite the evolutionary distance between dynein families, the shared organization and composition of the ODA complex with cytoplasmic dyneins suggests conserved regulatory mechanisms of activation. Cytoplasmic dyneins transition from an autoinhibited configuration characterized by crossed heavy chains to a functional state with parallel heavy chains^36,39^. While our structure of the ODA shows parallel β- and γ-HC tails, a recent structure of an inactive ODA-Shulin complex isolated from the cytoplasm of *Tetrahymena thermophila* cells shows a crossed conformation of the HC tails^49^. Therefore, the ODA complex must also transition from a crossed, inactive state to a parallel, active conformation when docking onto doublet microtubules, which is stabilized by association with the ODA-DC and inter-dynein contacts. For dynein-1, this transition is achieved by binding dynactin and coiled-coil adaptors^36,39^, but the factors regulating the activation of ODAs in cilia are currently unknown.

In summary, our structure provides insight into the microtubule-bound state of multi-headed axonemal dyneins and will serve as a reference to understand the mechanoregulatory mechanisms of ciliary motility.

## Methods

No statistical methods were used to predetermine the sample size. The experiments were not randomized and the investigators were not blinded to allocation during experiments and outcome assessment.

### *Chlamydomonas reinhardtii* culture and flagella isolation

*Chlamydomonas reinhardtii* (CC-1690) were cultured in liquid TAP media at room temperature under 12 h light/dark cycles in gyrating flasks. Flagella were isolated using a modified version of the dibucaine method^51^. Cells were harvested from 8 L of culture by centrifugation, washed twice with 10 mM HEPES pH 7.4, and resuspended in HMDS (10 mM HEPES, 5 mM MgSO_4_, 1 mM DTT, 4% sucrose, pH 7.4). Flagella were detached from cell bodies by treatment with 4.2 mM dibucaine and pipetting. Dibucaine treatment was quenched after 2-min incubation by the addition of HMDS containing 0.5 mM EGTA. Cell bodies were removed from detached flagella by pelleting the cell bodies with centrifugation at 2000 × *g* for 5 min. Detached flagella were further purified from residual cell bodies by underlaying the supernatant with a 25% sucrose cushion and centrifuging at 2500 × *g* for 10 min. The detached flagella in the supernatant were then concentrated by centrifugation at 3000 × *g* for 15 min.

### Preparing splayed axonemes

Freshly isolated flagella were resuspended in fresh HMDEKP (30 mM HEPES, 25 mM KCl, 5 mM MgSO_4_, 0.5 mM EGTA, 1× Protease Arrest (G-Biosciences)) to a concentration of 0.2–0.3 mg/mL and treated with 1% NP-40 for 30 min at 4 °C while rotating to remove the membrane. Axonemes were then washed to remove the detergent and soluble proteins by centrifuging at 2500 × *g* for 15 min and resuspending in HMDEKP. 0.5 mg/mL of axonemes were then splayed by incubation in HMDEKP containing 10 mM Mg^2+^ATP^2−^ and 750 μM CaCl_2_ for 1 h at room temperature while rotating.

### Negative-stain electron microscopy

4 μL of the splayed axoneme sample was adsorbed to glow-discharged, carbon-coated copper grids (Electron Microscopy Sciences, CF200-Cu) for 1 min before blotting with filter paper, and washed twice with 4 μL of 1.5% uranyl formate. The grids were then incubated with a third application of 4 μL 1.5% uranyl formate for 1 min, blotted, and air-dried before storing. Negative-stain grids were imaged using a Gatan UltraScan 894 (2k × 2k) CCD camera on a Phillips CM10 microscope equipped with a Tungsten filament operated at 100 kV.

### Cryo-EM sample preparation

Splayed axonemes were concentrated by centrifugation at 2500 × *g* and resuspended in cold HMDEKP to a concentration of 16–26 mg/mL. 2.5 μl of splayed axoneme solution was then dispensed onto glow-discharged C-Flat 1.2/1.3-4Cu grids inside a Vitrobot Mark IV under 100% humidity. Grids were glow-discharged on a Pelco easiGlow (Ted Pella) set to 15 mA for 30 s. After a 10 s delay time, cryo-EM samples were prepared by first blotting for 10 s with blot force set to 16 and immediately plunged into liquid ethane.

### Cryo-EM data collection

Cryo-EM samples were imaged on a Titan Krios Transmission Electron Microscope (ThermoFisher Scientific) operated a 300 kV at the Harvard Cryo-EM Center for Structural Biology. The microscope was equipped with a BioQuantum K3 Imaging Filter (slit width 25 eV) and a K3 direct electron detector (Gatan). A total of 20,524 images were recorded at a magnification of ×64,000, corresponding to a calibrated pixel size of 1.36 Å. A target defocus range of −0.5 to −2 μm was set during data collection. Images were dose-fractionated into 53 movie frames with a total exposure time of 3.7–3.8 s with a total dose of 61–62 electrons per Å^2^. Data collection utilized the SerialEM software^52^. A representative micrograph is shown in Supplementary Fig. 2a.

### Image processing

The overall strategy used to process the cryo-EM data is shown schematically in Supplementary Figs. 2 and 3. A total of 20,524 dose-fractionated image stacks were aligned and dose-weighted using MotionCor2 with 5 × 5 patches and a B-factor of 300^53^. Parameters of the contrast transfer function were estimated using CTFFIND4^54^. 18,361 micrographs were selected for further processing based on the absence of ice contamination and the quality of the Thon rings in the power spectra. Microtubules (including doublet microtubules and singlet microtubules) were manually picked on each micrograph by selecting their start and end coordinates in RELION-3.1-beta^55^. Overlapping sections of microtubules were avoided. The selected microtubule segments were computationally divided into overlapping boxes (512 × 512 pixels) with an 82 Å non-overlapping region (step size) between adjacent boxes, corresponding to the length of the tubulin α/β-heterodimer. We refer to these as “8-nm particles”. A total of 5,584,147 were extracted. Multiple rounds of 2D classification (Supplementary Fig. 2b) were used to discard particles that classified into poorly defined classes, resulting in a set of 5,163,521 particles for further processing.

The retained particles were subjected to one round of 3D refinement to determine their orientations, followed by a round of supervised classification using two references to separate the 8-nm particles into doublet and singlet microtubules. The references for supervised classification were the reported doublet microtubule (EMD-20631) and the proposed singlet microtubule, generated from the A tubule of a doublet microtubule^20^. Based on the classification result, 2,318,278 particles corresponding to doublet microtubules were kept for further refinement. To identify particles with bound ODAs, we first generated a generous binary mask over the ODA based on the subtomogram average of the *Chlamydomonas* axoneme^56^ (Supplementary Fig. 2c). Using this mask, we subtracted the signal from the doublet microtubule and performed 3D classification without alignment on just the ODAs (using 10 classes and regularization parameter *T* = 6). Classification yielded three classes with ODAs, corresponding to the three possible registers of the 8-nm particles within the 24-nm repeat. The two classes with off-centered ODA complexes were shifted 8 nm to be in register with the third class, and duplicate particles were eliminated using the Select function of RELION.

Upon manual examination of the particle locations in the raw micrographs, we realized that many ODA complexes were not retained in previous rounds of 3D classification. We, therefore, devised a strategy to recover more particles by using our known coordinates to select neighboring ODA complexes. Since ODA complexes are retained in clusters and repeat every 24 nm, new particles were extracted ±24 and 48 nm along the microtubule axis relative to the particles obtained through 3D classification, and combined with the original particles. After elimination of duplicates, the remaining particles were subject to masked refinement on the ODA complex and underlying protofilaments A06–08 to compel particle alignment with the same 8-nm register. Following this step, the particles were re-extracted to enforce the correct 8-nm register and underwent unmasked refinement. The refined maps were subject to masked 3D classification without alignment on the ODAs and underlying protofilaments A06–08 (using 2 classes and regularization parameter *T* = 6). The selected class with high ODA density was then checked by manual examination of particle locations on the micrographs to verify the presence of ODA complexes and correct 24 nm spacing between particles. This procedure was repeated and increased our particle number from 282,069 to 485,694 particles with bound ODA complexes. Next, the retained particles were re-extracted to center the ODA complex within a box of 550 pixels and underwent CTF refinement and particle polishing.

After refinement of the polished particles, signal subtraction was performed to yield a smaller box size of 420 pixels instead of 550 for more focused refinement and faster processing. Refinement of the subtracted particles yielded a 3.8-Å map. Additional signal subtractions and mask-focused refinements were used to obtain higher-resolution maps of ODA subcomplexes suitable for model building (Supplementary Fig. 3c).

### Generation of composite maps

The structure is deposited as three composite maps corresponding to (1) the complete ODA, (2) the ODAcore consisting of the IC–LC block and heavy chain tails, and (3) the ODA-DC consisting of protofilaments A07-A08, the DC1/DC2 coiled-coil and DC3 (Supplementary Table 1). The composite maps were generated by merging mask-focused refinements using the vop maximum command in Chimera.

### Model building

Before starting model building, homology models were generated using I-TASSER^57^ and SWISS-MODEL^58^. Protein sequences were obtained from Phytozome v13 except for γ-HC, which was obtained from UniProt. The database accession numbers of all sequences used are given in Supplementary Table 2.

Homology models for the light chains were generated using either I-TASSER (LC7a, LC7b) or SWISS-MODEL (LC3, LC4, LC5 LC6, LC8, LC9, LC10). Homodimers were built for all light chains except LC3-LC5. The IC–LC block contains density for one dimer of the roadblock/LC7 family, one dimer of TCTEX-like domains, and three dimers of the LC8 family. Initially, we assumed each dimer corresponded to a homodimer, so we placed homodimers of LC2, LC6, LC7a, LC7b, LC8, LC9, and LC10 into each of the possibilities using the fit-to-map procedure in Chimera^59^ or Jiggle Fit in Coot^60^. Comparison of the densities with the models revealed unambiguously that LC7a exists as a heterodimer with LC7b (Supplementary Fig. 4a). This is consistent with reports that Roadblock/LC7 family members form heterodimers in vitro^61^ and in structures of other complexes^62^. Additionally, the density shows that LC8 forms heterodimers with LC6 and LC10, as well as homodimers. LC10 could be distinguished from its paralogs based on the presence of an N-terminal helix absent from either LC6 or LC8. LC6 could be distinguished from LC8 and LC10 based on the presence of an elongated loop between the α2 helix and the β2 strand (Supplementary Fig. 4b). Based on the presence of multiple heterodimers in the structure of the ODA, we assume that the TCTEX-like domains LC2 and LC9 also form a heterodimer, although the density is insufficiently resolved to make a conclusive assignment. A heterodimer of LC2/LC9 is consistent with previous crosslinking data that positions both LC2 and LC9 at the IC–LC block; LC2 crosslinks with LC6, and LC9 crosslinks with IC1 and IC2^63,64^. LC4 is monomeric and was positioned in the map using Jiggle Fit^60^. Where sidechains were resolved, the models were manually adjusted to better fit the density in Coot v0.9. Where sidechains were not resolved, the model has been pruned to the C_β_ position to signify the uncertainty.

Homology models for the intermediate chains IC1 and IC2 were generated using I-TASSER, placed in the density using Jiggle Fit in Coot^60^, and manually adjusted to better fit the density in Coot v0.9. Termini and loops not well predicted by the homology models were built de novo.

The N-terminal dimerization domains (NDD) of β-HC and γ-HC were modeled in I-TASSER and placed into density in Coot. The helical bundles were initially modeled as a series of idealized α-helices. The orientation and connectivity of the helices was guided by the model of the heavy chain of human dynein-2^37^. The sequence was assigned based on secondary structure profiles determined using JPred^65^. For some regions, the density was sufficiently resolved to place sidechains (Supplementary Fig. 4). For other regions where the registry is less certain, we have left the sidechains unmodeled. Loops connecting the α-helices were built manually using Coot. Homology models for the AAA+ motor domains and associated linker regions of all three heavy chains were generated using SWISS-MODEL and placed into the density using the fit-to-map procedure in Chimera. A model for the N-terminal six-bladed β-propeller of α-HC was generated in SWISS-MODEL and placed in the density using Jiggle Fit^60^.

The partial model of the ODA-DC^20^ was docked into the density in Chimera. The C-terminal half of the DC1/DC2 coiled-coil was built as idealized α-helices in Coot with an uncertain registry. The C-terminal region of DC3 was built de novo in Coot.

### Refinement

The homology models of the AAA+ motor domains and linker regions of α-HC, β-HC, and γ-HC were fit to the density using molecular dynamics flexible fitting and real-space refinement implemented through the Namdinator pipeline^66^. The β-HC and γ-HC models were then further refined using Phenix.real_space_refinement v.1.18.2^67^. During the refinement of the motor domains, the resolution limit was set to match the resolution of the maps determined using the FSC = 0.143 criterion (4.5 Å for γ-HC and 6.2 Å for β-HC). Reference restraints from PDB 5NUG^39^ were used, as were secondary structure, Ramachandran and rotamer restraints. Sidechains were then truncated and model statistics calculated using phenix.molprobity^68^. Model statistics are not calculated for α-HC as the resolution is worse than 10 Å.

The atomic models of the ODA-DC (consisting of protofilaments A07-A08 and DC1/DC2/DC3) and ODAcore (consisting of the heavy chain tail domains and IC–LC block) were refined against their relevant composite maps using real-space refinement in Phenix^67^. Secondary structure, Ramachandran, and rotamer restraints were used and the resolution limit set to 3.6 Å. A single round of simulated annealing was performed midway through the macrocycle. Rounds of manual model correction in Coot^60^ were performed between rounds of refinement in Phenix. During real-space refinement in Coot, torsion, planar peptide, and Ramachandran restraints were used. The final models were validated using phenix.molprobity^68^. Model statistics are provided in Supplementary Table 3.

### Figures

Figure panels were generated using Chimera^59^ or ChimeraX^69^. Maps colored by local resolution were generated using RELION-3.1-beta^55^.

Software used in the project were installed and configured by SBGrid^70^.

### Reporting summary

Further information on research design is available in the Nature Research Reporting Summary linked to this article.

## Supplementary information

Supplementary Information

Supplementary Movie 1

Supplementary Movie 2

Supplementary Movie 3

Reporting Summary

## Data Availability

Data supporting the findings of this manuscript are available from the corresponding author upon reasonable request. A reporting summary for this Article is available as a Supplementary Information file. Composite cryo-EM maps have been deposited in the Electron Microscopy Data Bank (EMDB) with accession codes 23082, 23083, and 23084 corresponding to the complete ODA, the ODAcore, and the ODA-DC, respectively. Constituent maps and the masks that were applied during processing are deposited as additional files (listed in Supplementary Table 1). Atomic models have been deposited in the Protein Data Bank (PDB, https://www.rcsb.org/) with accession codes 7KZM, 7KZN, and 7KZO. Source data are provided with this paper.

## Source data

Source Data
