# Supplementary material for: Structure of a microtubule-bound axonemal dynein
Source: Nat Commun. 2021 Jan 20;12:477. doi: 10.1038/s41467-020-20735-7 (PMC7817835; doi:10.1038/s41467-020-20735-7)
Supplement: Supplementary file 1 — Supplementary Information [file 41467_2020_20735_MOESM1_ESM.pdf]

Supplementary Information

Structure of a microtubule-bound axonemal dynein

Travis Walton, Hao Wu, Alan Brown

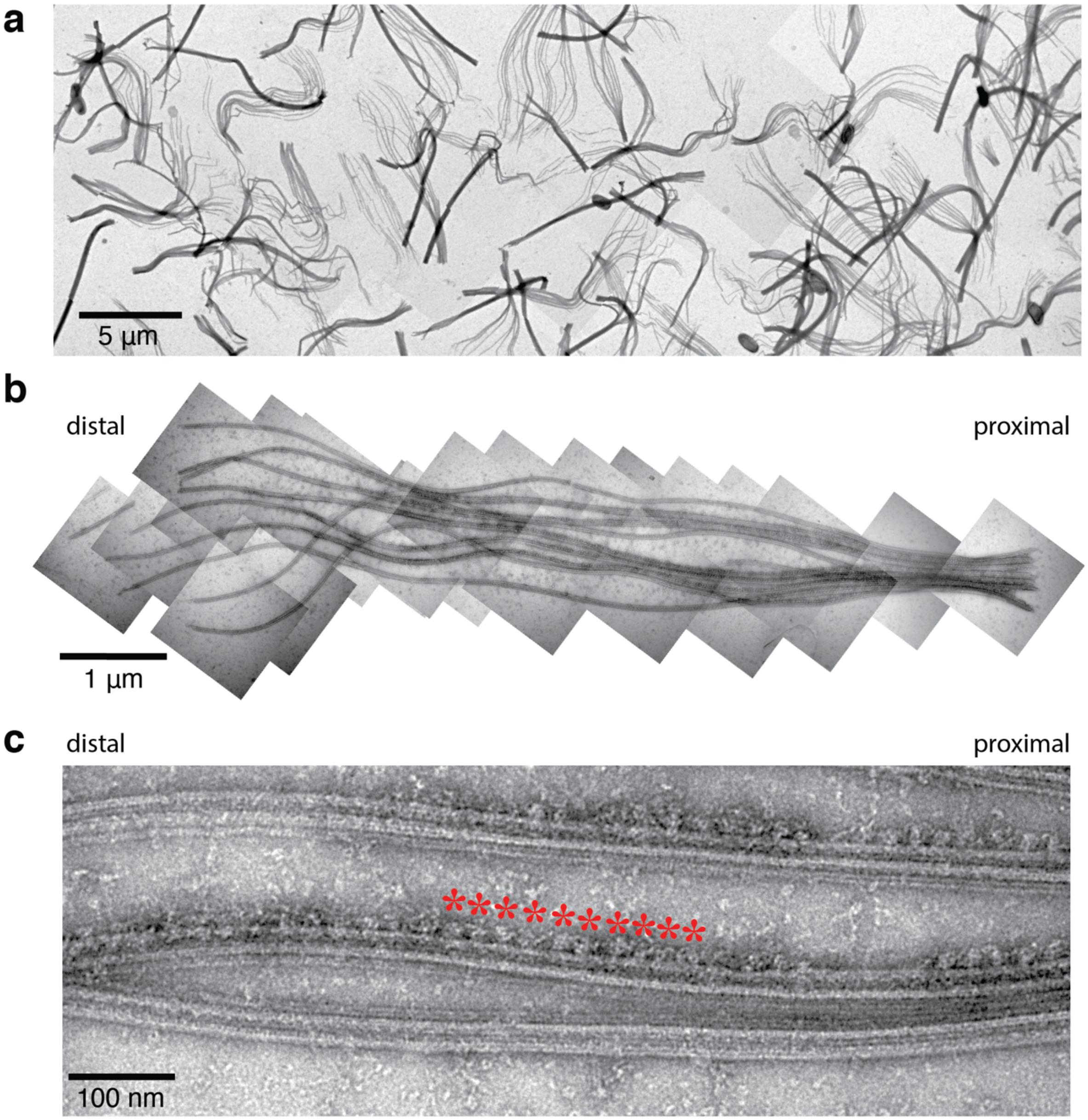

Supplementary Fig. 1 | Negative-stain electron microscopy showing that axonemes splayed with ATP retain ODA complexes. a, Low-magnification composite of axonemes after incubation with 10 mM ATP for 1 hour. Fraying, splaying, and complete dissociation of axonemes can be observed. **b**, Collage of micrographs covering an entire splayed axoneme. The proximal end of the axoneme is still loosely associated, while the distal end (identified by the transition of doublet microtubules to singlet microtubules) is completely splayed apart. **c**, Close-up view of single micrograph showing doublet microtubules bound by axonemal complexes (denoted by red asterisks). The 24-nm periodicity and shape of these complexes are consistent with ODAs. Micrographs in panel A were taken at 1540× magnification. Micrographs in panels B and C were taken at 21,700×.

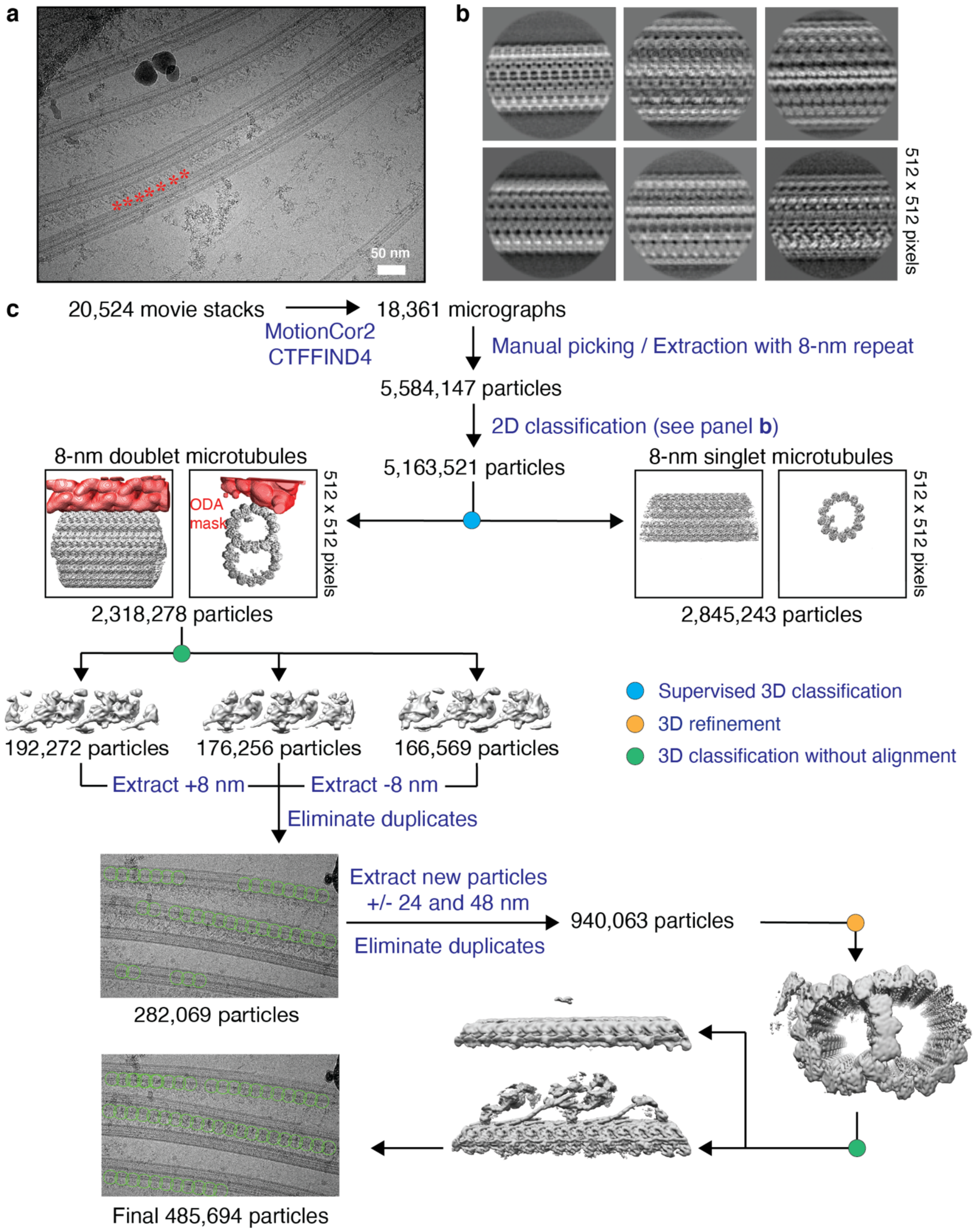

Supplementary Fig. 2 | Data processing strategy. a, Representative micrograph showing purified doublet microtubules. Examples of ODA complexes bound to doublet microtubules are denoted by red asterisks. **b**, Selected two-dimensional class averages. **c**, Processing scheme used to select 24-nm sections of doublet microtubules with bound ODA complexes.

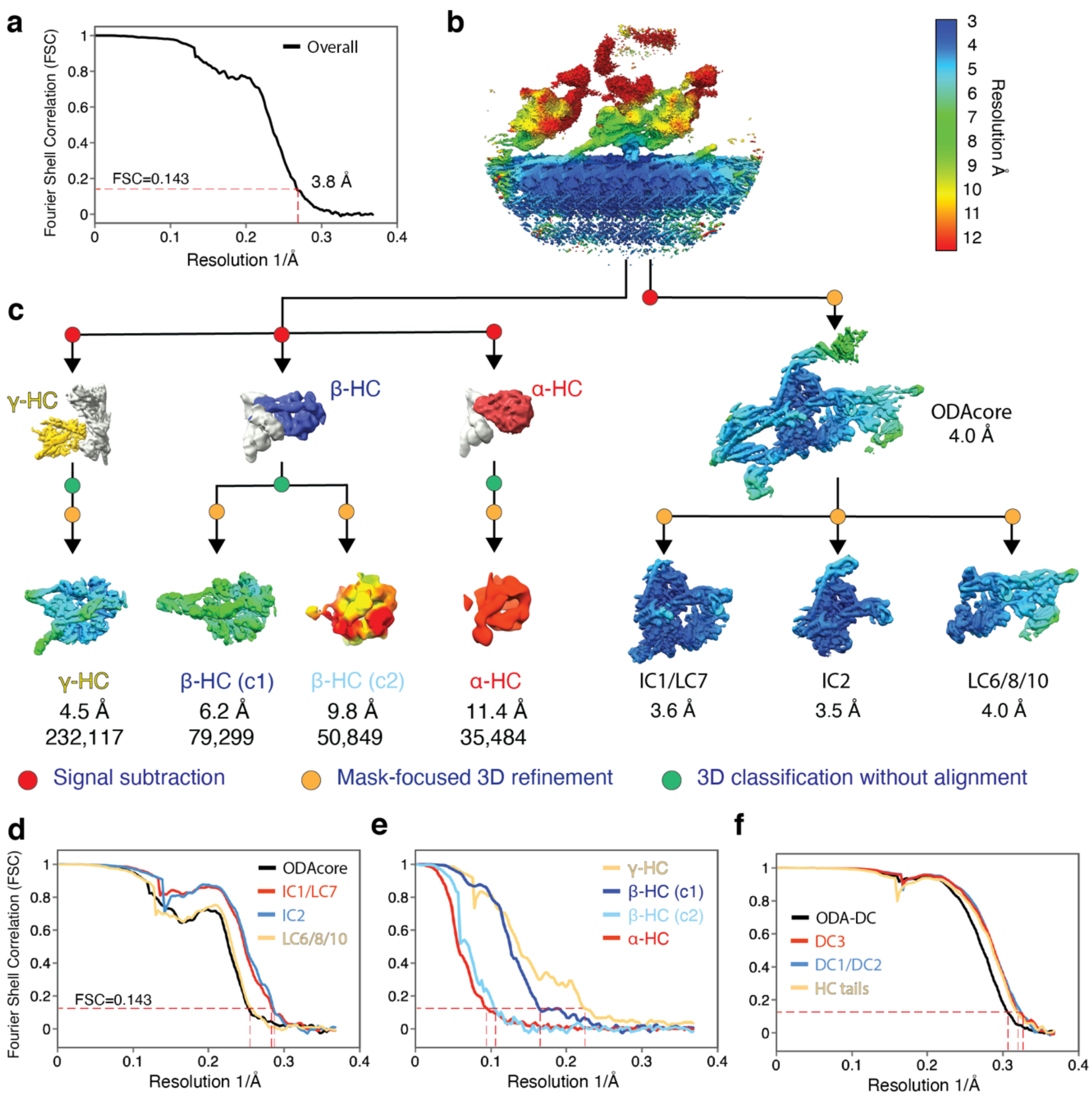

Supplementary Fig. 3 | Global and local resolution. a, Fourier shell correlation (FSC) curve calculated between masked independent half maps for the microtubule-bound ODA. The nominal resolution is 3.8 Å, estimated using the FSC = 0.143 criterion (red dashed line). **b**, Density map for the microtubule-bound ODA colored by local resolution. The local resolution is colored from 3 to 12.5 Å. **c**, Processing scheme used to improve the map quality of local regions of the ODA complex. Two different conformations of the β-HC motor domain were identified (c1 and c2). **d**, FSC curves calculated between masked independent half maps for the core of the ODA and its subregions. **e**, FSC curves calculated between masked independent half maps for the motor domains of the three heavy chains. **f**, FSC curves calculated between masked independent half maps for the ODA docking complex (ODA-DC). In panels d-f, the dashed red lines indicate the resolution at FSC = 0.143.

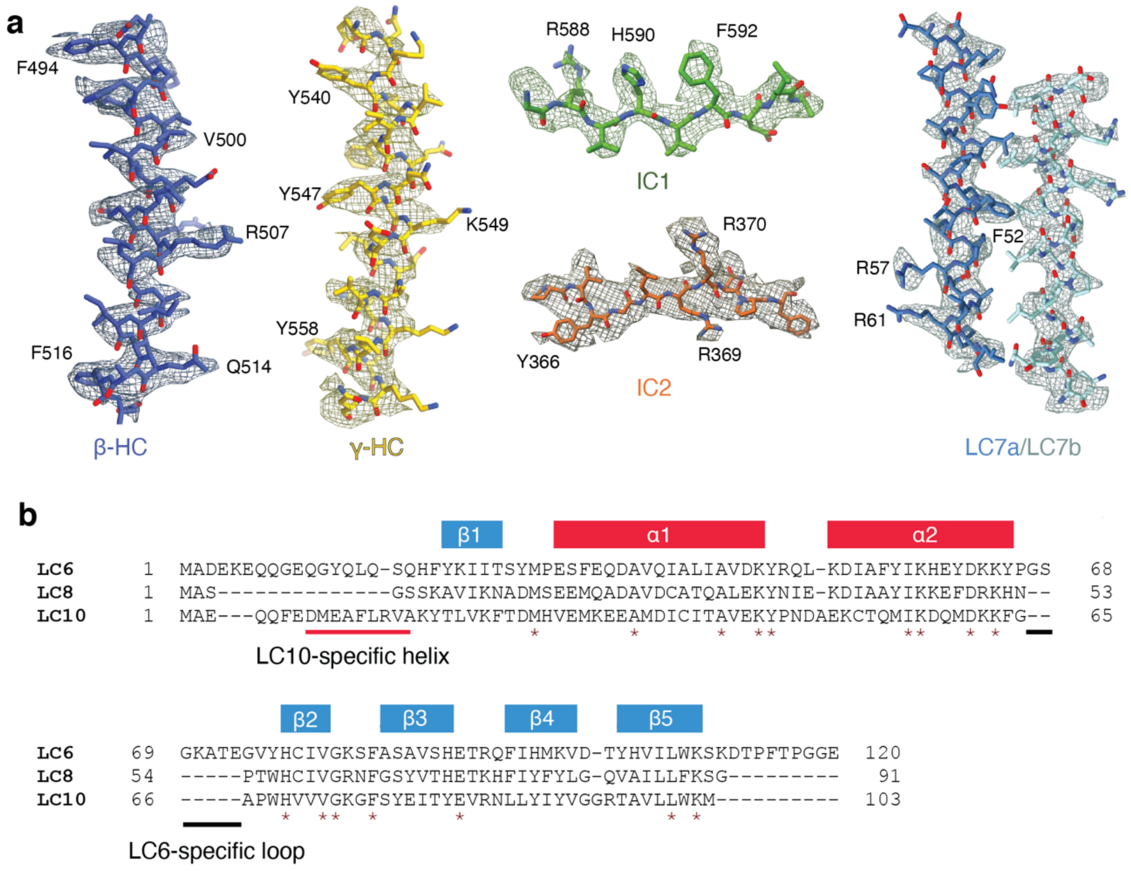

Supplementary Fig. 4 | Map quality and model building. a, Examples of regions of the map with resolved sidechains. The density maps are contoured at 0.014-0.02. Landmark residues are labeled. The resolved sidechains clearly distinguish LC7a from LC7b. **b**, Sequence alignment of LC6, LC8 and LC10 with secondary structure annotation. LC10 could be distinguished from its paralogs based on the presence of an N-terminal helix absent from either LC6 or LC8. LC6 could be distinguished from LC8 and LC10 based on the presence of an elongated loop between the α2 helix and the β2 strand.

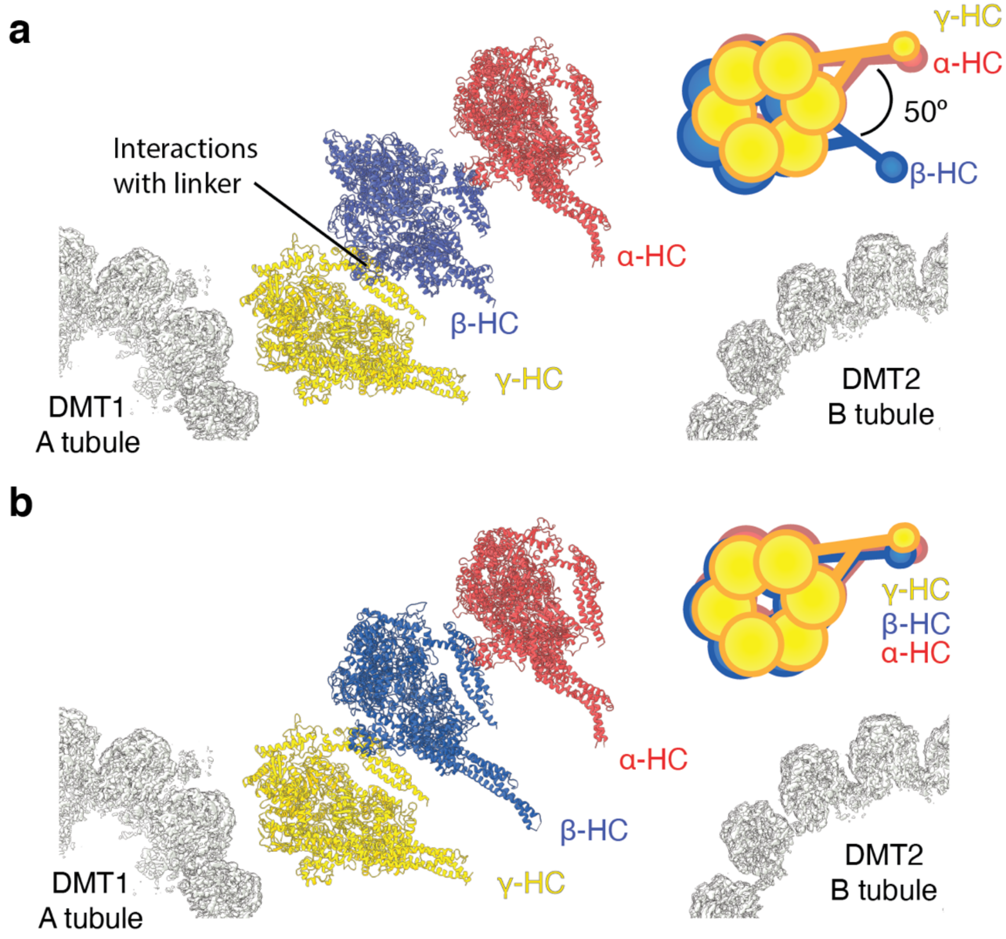

Supplementary Fig. 5 | Orientations and interactions of the motor domains. a, The orientation of the motor domains with respect to the doublet microtubule to which they are docked (DMT1) and the doublet microtubule to which they transiently interact (DMT2). The doublet microtubules maps are from EMD-20631[1] and are positioned using a subtomogram average volume of the *C. reinhardtii* axoneme which contains two neighboring doublet microtubules (EMD-20341)[2]. The β-HC motor domain interacts with the linker of γ-HC in this conformation. The schematic (top right corner) shows the relative orientation of the stalk and microtubule-binding domain when viewed from below the γ-HC motor. The stalk of β-HC is rotated ~50° relative to the stalks of α- and γ-HC. **b**, Orientation of the motor domains in the alternative class of the β-HC motor domain (class 2). The stalks all point in the direction of the B tubule of DMT2 and overlap when viewed from below the γ-HC motor (schematic).

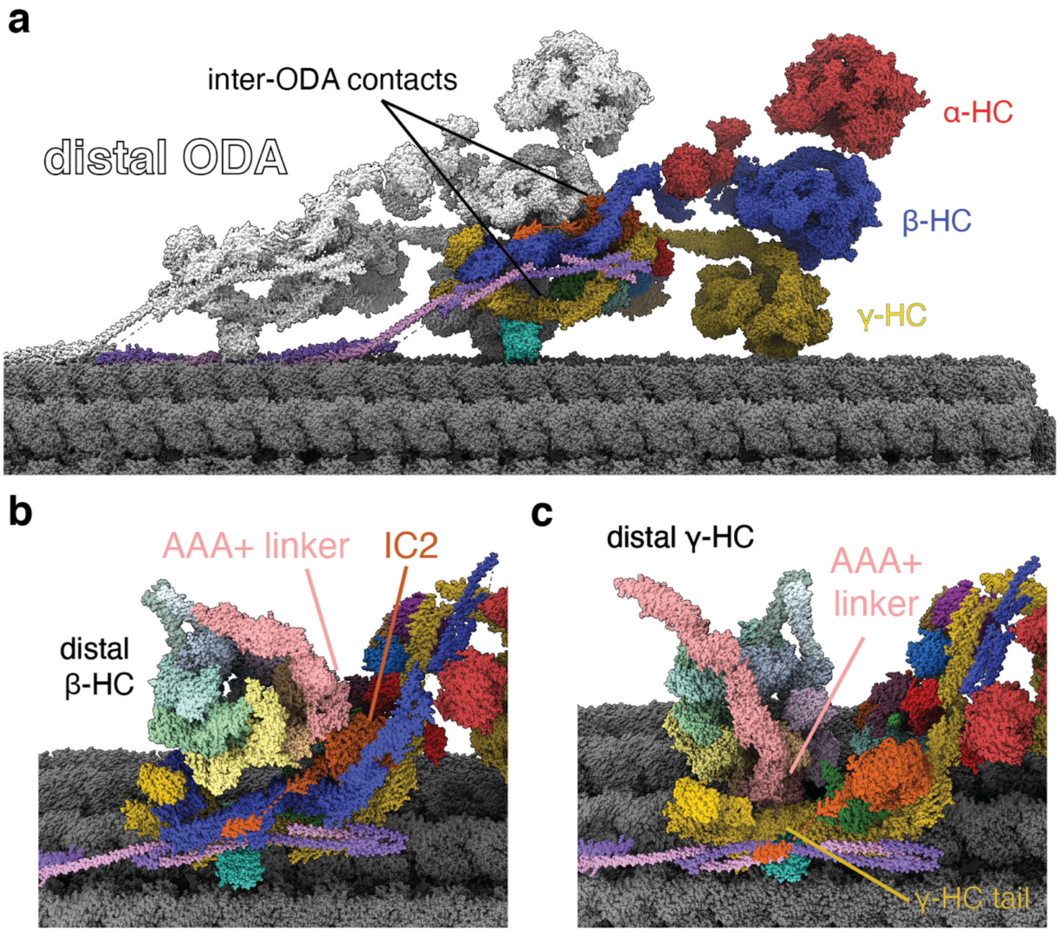

Supplementary Fig. 6 | The slanted, overlapping conformation of doublet microtubule-bound ODAs causes interaction between adjacent complexes. a, The N-terminal tails of the β- and γ-HCs (blue and yellow) envelop the AAA+ domains of its distal ODA neighbor (white). **b**, The β-HC of the distal ODA complex interacts with IC2 (orange) of the IC-LC complex through the linker region (light red) of its AAA+ domain. **c**, Underneath the distal β-HC, the AAA+ domain linker region (light red) of the distal γ-HC interacts with the N-terminal tails of the neighboring γ-HC (yellow).

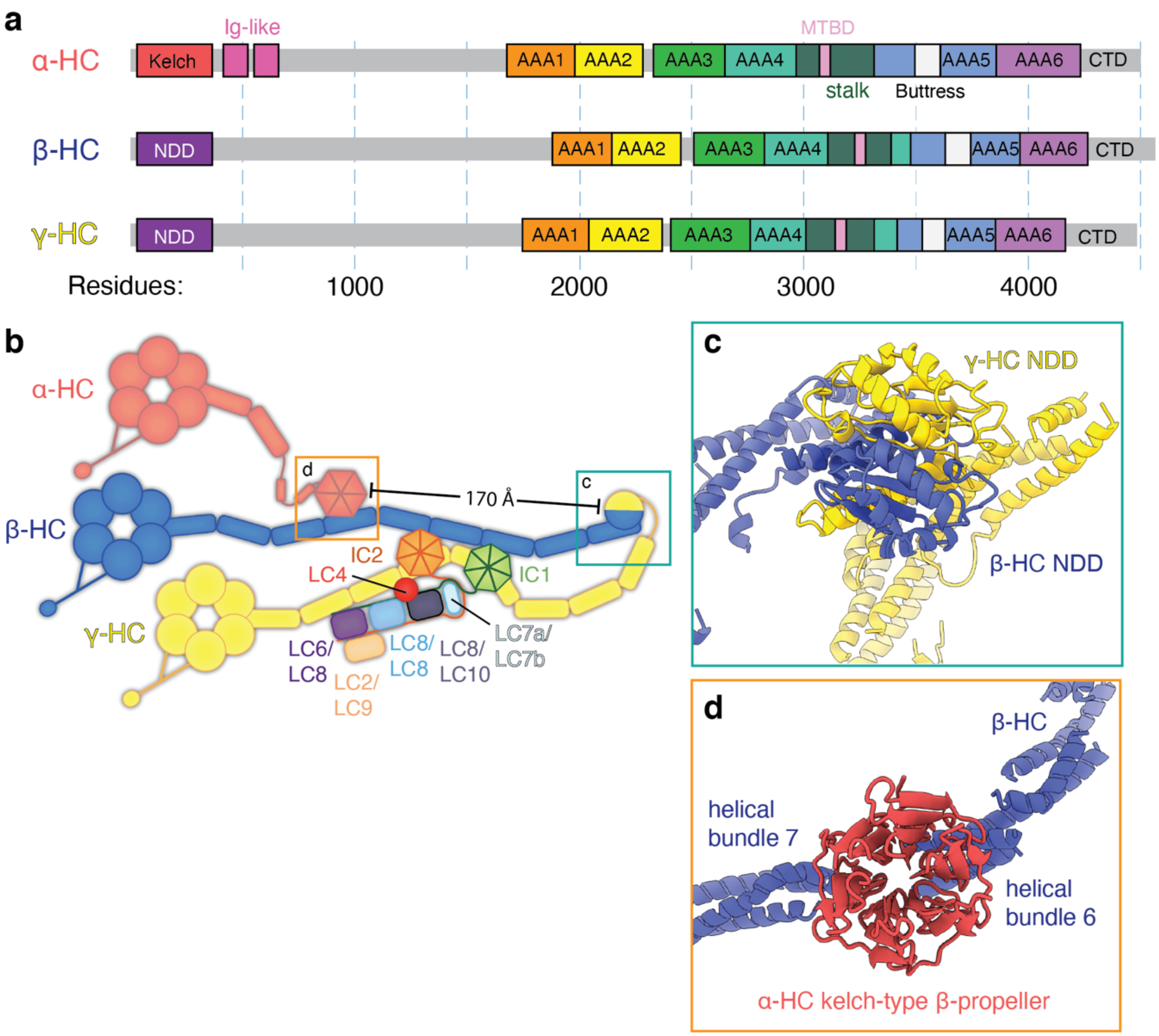

Supplementary Fig. 7 | Heterotrimerization of the ODA heavy chains. a, Domain architecture of the heavy chains of the *C. reinhardtii* ODA. **b**, Cartoon of the architecture of the ODA. The boxed sections are shown in panels c and d. **c**, Atomic model showing heterodimerization of β- and γ-HC through their N-terminal dimerization domains (NDD). **d**, Atomic model showing the interaction between the kelch-type β-propeller of α-HC with helical bundles 6 and 7 of the β-HC tail.

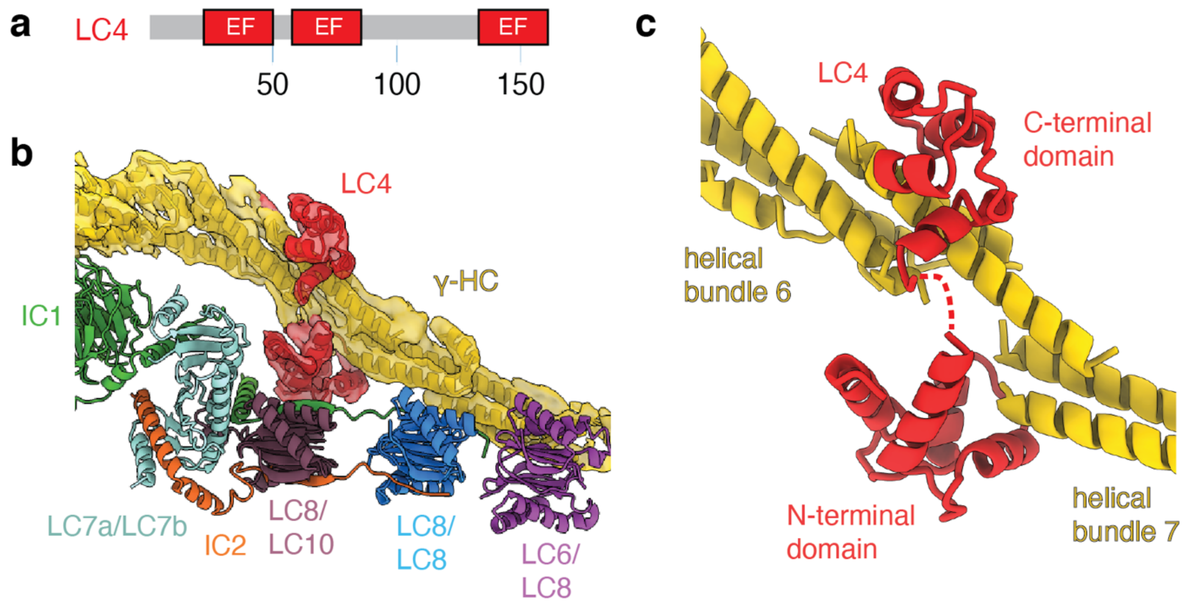

Supplementary Fig. 8 | LC4 interacts with the helical bundles of the γ-HC. a, Domain architecture of LC4. The N-terminal domain contains two EF-hand motifs and the C-terminal domain contains one EF-hand motif. **b**, Molecular environment of LC4. Atomic models are shown for all subunits, with transparent density maps for LC4 and γ-HC. **c**, Atomic model of the interaction between LC4 and the junction between helical bundles 6 and 7 of the γ-HC. Loops at the junction are unmodeled due to flexibility.

Supplementary Table 1 | Deposited composite maps and their associated maps. Postprocessed maps, both half maps and the binary mask used for focused refinement and postprocessing have been included for every deposited composite map.

Entry	EMDB code	Associated maps
**ODA composite**	EMD-23082	Full ODA map (consensus refinement)<br>γ-HC AAA+ map<br>α-HC AAA+ map<br>α-HC tail map<br>β-HC AAA+ map (conformation 1)<br>β-HC AAA+ map (conformation 2)<br>γ-HC AAA+ map
**ODAcore composite**	EMD-23083	ODAcore (consensus refinement)<br>IC1/LC7a<br>IC2<br>LC6/8/10
**ODA-DC composite**	EMD-23084	ODA-DC (consensus refinement)<br>DC1/DC2<br>DC3<br>β-HC and γ-HC N-terminal tails

Supplementary Table 2 | Subunits of the *C. reinhardtii* ODA and model accuracy.

Protein	Phytozome accession	Mass (Da)	Model accuracy	Notes
α-HC	Cre03.g145127.t1.1	503,617	Fold (Tail & Motor)	
β-HC	Cre09.g403800.t1.2	519,972	Sidechain (Tail)<br>Fold (Motor)	
γ-HC	Q39575 (UniProt)	512,846	Sidechain (Tail)<br>Fold (Motor)	
IC1	Cre12.g536550.t1.2	76,525	Sidechain	
IC2	Cre12.g506000.t1.2	63,520	Sidechain	
LC1	Cre02.g092850.t1.2	22,151	Not observed	Binds MTBD of γ-HC
LC2	Cre12.g527750.t1.2	15,883	Fold	Potentially forms a heterodimer with LC9
LC3	Cre12.g528850.t1.1	17,365	Not observed	
LC4	Cre01.g051250.t1.2	17,788	Fold	
LC5	Cre17.g714250.t1.2	14,179	Not observed	
LC6	Cre03.g187200.t1.2	13,857	Fold	Forms a heterodimer with LC8
LC7a	Cre08.g376550.t1.2	11,928	Sidechain	Forms a heterodimer with LC7b
LC7b	Cre12.g546400.t1.2	11,119	Sidechain	Forms a heterodimer with LC7a
LC8	Cre03.g181150.t1.1	10,322	Fold, some sidechains	Forms heterodimers with LC6 and LC10, as well as a homodimer
LC9	Cre10.g428850.t1.2	12,668	Fold	Potentially forms a heterodimer with LC2
LC10	Cre12.g527800.t1.1	12,086	Fold, some sidechains	Forms a heterodimer with LC8
DC1	Cre17.g703850.t1.2	83,381	Sidechain (N-terminus)	
DC2	Cre16.g666150.t1.2	62,204	Sidechain (N-terminus)	
DC3	Cre14.g617550.t1.2	21,341	Sidechain	

Supplementary Table 3 | Statistics for data collection, data processing, model refinement and validation.

**Data collection**				
Microscope model	Titan Krios (Thermo Fisher)			
Detector model	K3 (Gatan)			
Voltage (keV)	300			
Nominal magnification	64,000 ×			
Electron exposure ($e^-/Å^2$)	60			
Target defocus range (μm)	-0.5 to -2.0			
Pixel size (Å)	1.36			
Number of datasets	3			
Micrographs collected	20,524			
**Data Processing**				
Micrographs used for particle picking	18,361			
Final particle number	485,694			
Map resolution range (Å)	3.7 - 12			
**Model composition**	**ODAcore (HC tails and IC–LC block)**	**ODA-DC**	**γ-HC motor domain**	**β-HC motor domain**
Chains	19	20	1	1
Atoms	31,213	54,115	14,462	14,136
Residues	4,099	6,873	2,925	2,857
Ligands	0	7 GTP; 7 GDP, 7 Mg	0	0
**Refinement**				
Resolution limit set in refinement (Å)	3.6	3.6	4.5	6.2
Correlation coefficient (CCmask)	0.67	0.76	0.73	0.68
Root-mean-square deviation (bond lengths) (Å)	0.010	0.005	0.003	0.005
Root-mean-square deviation (bond angles) (Å)	1.354	0.852	0.766	1.069
B factors (Protein) ($Å^2$)	90.6	65.6	137.4	460.4
**Validation**				
MolProbity Score	2.1	1.9	1.8	2.0
Clash score	10.7	13.8	4.6	7.3
Rotamer outliers (%)	0.5	0.3	0	0
Ramachandran (favored) (%)	89.8	96.0	90.1	87.8
Ramachandran (outliers) (%)	0.2	0.03	0.03	0.04
PDB code	7KZN	7KZO	7KZM	

Supplementary References

1. Ma, M. et al. Structure of the decorated ciliary doublet microtubule. *Cell* **179**, 909–922.e12 (2019).

2. Gui, L. et al. Scaffold subunits support associated subunit assembly in the Chlamydomonas ciliary nexin-dynein regulatory complex. *Proc. Natl Acad. Sci. USA* **110**, 2 01910960 (2019).
