# Supplementary material for: Structure of a microtubule-bound axonemal dynein
Source: Nat Commun. 2021 Jan 20;12:477. doi: 10.1038/s41467-020-20735-7 (PMC7817835; doi:10.1038/s41467-020-20735-7)
Supplement: Supplementary file 5 — Reporting Summary [file 41467_2020_20735_MOESM5_ESM.pdf]

nature research

Corresponding author(s): Alan Brown

Last updated by author(s): Dec 7, 2020

Reporting Summary

Nature Research wishes to improve the reproducibility of the work that we publish. This form provides structure for consistency and transparency in reporting. For further information on Nature Research policies, see our Editorial Policies and the Editorial Policy Checklist.

Statistics

For all statistical analyses, confirm that the following items are present in the figure legend, table legend, main text, or Methods section.

n/a	Confirmed	
☒	☐	The exact sample size (*n*) for each experimental group/condition, given as a discrete number and unit of measurement
☒	☐	A statement on whether measurements were taken from distinct samples or whether the same sample was measured repeatedly
☒	☐	The statistical test(s) used AND whether they are one- or two-sided<br>*Only common tests should be described solely by name; describe more complex techniques in the Methods section.*
☒	☐	A description of all covariates tested
☒	☐	A description of any assumptions or corrections, such as tests of normality and adjustment for multiple comparisons
☒	☐	A full description of the statistical parameters including central tendency (e.g. means) or other basic estimates (e.g. regression coefficient) AND variation (e.g. standard deviation) or associated estimates of uncertainty (e.g. confidence intervals)
☒	☐	For null hypothesis testing, the test statistic (e.g. *F*, *t*, *r*) with confidence intervals, effect sizes, degrees of freedom and *P* value noted<br>*Give P values as exact values whenever suitable.*
☒	☐	For Bayesian analysis, information on the choice of priors and Markov chain Monte Carlo settings
☒	☐	For hierarchical and complex designs, identification of the appropriate level for tests and full reporting of outcomes
☒	☐	Estimates of effect sizes (e.g. Cohen's *d*, Pearson's *r*), indicating how they were calculated

Our web collection on statistics for biologists contains articles on many of the points above.

Software and code

Policy information about availability of computer code

Data collection	Cryo-EM datasets were collected with SerialEM v.3.6 and v.37. Negative stain EM data were collected using Digital Micrograph v. 3.9.4. Protein sequences were collected from Phytozome v.13 and UniProt release 2020_02.<br>Homology models were generated using I-TASSER (https://zhanglab.ccmb.med.umich.edu/I-TASSER, no version number) and SWISS-MODEL (https://swissmodel.expasy.org/, no version number).
Data analysis	MotionCor2; CTFFIND4; RELION-3.1-beta; Chimera v.1.14; Namdinator (https://namdinator.au.dk, no version number), Coot v0.8.9.2; Coot v0.9; phenix.real_space_refine v1.18.2, Phenix.molprobity v1.18.2; ChimeraX v1.0

For manuscripts utilizing custom algorithms or software that are central to the research but not yet described in published literature, software must be made available to editors and reviewers. We strongly encourage code deposition in a community repository (e.g. GitHub). See the Nature Research guidelines for submitting code & software for further information.

Data

Policy information about availability of data

All manuscripts must include a data availability statement. This statement should provide the following information, where applicable:

- Accession codes, unique identifiers, or web links for publicly available datasets
- A list of figures that have associated raw data
- A description of any restrictions on data availability

Composite cryo-EM maps have been deposited in the Electron Microscopy Data Bank (EMDB; https://www.ebi.ac.uk/pdbe/emdb/) with all constituent maps (and the masks that were applied) deposited as additional files. EMDB accession codes are 23082, 23083, and 23084. Atomic models have been deposited in the Protein Data Bank (PDB; https://www.rcsb.org/) with codes 7KZM, 7KZN, and 7KZO.

Field-specific reporting

Please select the one below that is the best fit for your research. If you are not sure, read the appropriate sections before making your selection.

☒ Life sciences ☐ Behavioural & social sciences ☐ Ecological, evolutionary & environmental sciences

For a reference copy of the document with all sections, see nature.com/documents/nr-reporting-summary-flat.pdf

Life sciences study design

All studies must disclose on these points even when the disclosure is negative.

Sample size	No statistical methods were used to predetermine sample size.<br>Negative stain electron microscopy data were collected in sufficient amounts to create a montage showing a complete, splayed axoneme.<br>Cryo-EM data were collected in sufficient amounts to achieve adequate resolution reconstructions for the outer dynein arm.
Data exclusions	In the cryo-EM images analysis process, particles are grouped by the Relion software into multiple 2D and 3D classes. 2D and 3D classes that did not contribute to a high-resolution 3D density map of the outer dynein arms were excluded.
Replication	Replication was not performed to explicitly test reproducibility. However, processing datasets from different data collections resulted in similar reconstructions.
Randomization	Reconstructions used two randomized half sets to prevent over-refinement of the map and to assess the resolution of the final reconstruction. Randomizaton serves no purpose in other analyses.
Blinding	Blinding was not used because this is a study of molecular structure. Blinding would serve no purpose.

Reporting for specific materials, systems and methods

We require information from authors about some types of materials, experimental systems and methods used in many studies. Here, indicate whether each material, system or method listed is relevant to your study. If you are not sure if a list item applies to your research, read the appropriate section before selecting a response.

Materials & experimental systems

n/a	Involved in the study
☒	☐ Antibodies
☒	☐ Eukaryotic cell lines
☒	☐ Palaeontology and archaeology
☒	☐ Animals and other organisms
☒	☐ Human research participants
☒	☐ Clinical data
☒	☐ Dual use research of concern

Methods

n/a	Involved in the study
☒	☐ ChIP-seq
☒	☐ Flow cytometry
☒	☐ MRI-based neuroimaging
